# The Prognostic Value of PARP Expression in High-Grade Epithelial Ovarian Cancer

**DOI:** 10.1007/s12253-020-00856-6

**Published:** 2020-06-27

**Authors:** Szabolcs Molnár, Lívia Beke, Gábor Méhes, Róbert Póka

**Affiliations:** 1 Institute of Obstetrics and Gynaecology, University of Debrecen, Nagyerdei krt. 98, Debrecen, 4032 Hungary; 2 Institute of Pathology, University of Debrecen, Nagyerdei krt. 98, Debrecen, 4032 Hungary

**Keywords:** Ovarian cancer, PARP expression, Gynaecological oncology, Platinum-based chemotherapy, Progression-free survival

## Abstract

In an attempt to clarify the prognostic relevance of poly (ADP-ribose) polymerase (PARP) expression, we analysed the clinical data of 86 high-grade epithelial ovarian cancer (EOC) cases in which PARP immunohistochemistry results were available. Immunostaining to highlight PARP protein expression was performed using a Leica Bond MAX Immunostainer (Leica Microsystems, Wetzlar, Germany). We applied a rabbit polyclonal anti-PARP antibody (ab6079 330, Abcam, Cambridge, UK) for the specific reaction. The intensity and distribution of immunostaining were assessed by light microscopy (Leica DM2500 microscope, DFC 420 camera, and Leica Application Suite V3 software; Leica) and evaluated with a four-grade (0–3+) system. The median progression-free survival (PFS) was generated for each semiquantitative group of PARP expression among chemotherapy-naive cases at the time of PARP immunohistochemistry. Eighty-six cases were chemotherapy-naive at the time of PARP immunohistochemistry, and 41 cases showed no PARP expression. Forty-five cases showed intermediate or high PARP expression. The median PFS among patients in the PARP-negative group was 16 months (interquartile range; IQR 10.7–35.9 months), and the median PFS of patients in the PARP-positive group was 12 months (IQR 6.1–21.8 months). The difference was significant according to the log-rank test (*p* = 0.01). The median overall survival (OS) of patients in the PARP-negative group was 65 months (IQR 43.6–110.8 months), and the median OS of patients in the PARP-positive group was 52 months (IQR 36.9–66.7 months). The difference was significant according to the log-rank test (*p* = 0.028). Multiple comparisons confirmed that PARP expression results in a significant difference in PFS and OS achieved by first-line Taxol-carboplatin chemotherapy. The lack of PARP expression assessed by immunohistochemistry may predict improved PFS in ovarian cancer patients after adjuvant platinum-based chemotherapy.

## Introduction

Ovarian cancer has the highest mortality among female genital cancers, and it is the seventh most significant contributor to cancer mortality [1, 2]. Nearly 75% of ovarian cancer cases present in advanced stages [3]. Epithelial ovarian cancer (EOC) has a unique tumour biology and pattern of metastasis [4]. However, according to recent studies, most of these tumours originate from a lesion in the distal fallopian tube and are termed serous tubal intraepithelial carcinomas (STICs). Immunohistochemical staining for the p53 protein is positive in a significant proportion of these lesions, similar to high-grade serous ovarian cancer [4, 5].

.The effect of conventional anticancer therapies is damage to nuclear DNA and chromosomal integrity. These treatments primarily destroy cancer cells and reduce their survival capacity. Poly adenosine diphosphate (ADP)-ribose polymerase (PARP) inhibition is one of the latest therapeutic approaches for the treatment of ovarian cancer. PARP is part of an alternative DNA repair mechanism and plays an essential role in detecting and initiating the cellular response to single-strand breaks (SSBs) by signalling the enzymatic repair mechanism. PARP binds to deficient segments of DNA and begins the synthesis of a poly (ADP-ribose) (PAR) chain. BRCA mutations are one of the most common hereditary genetic defects that occur in ovarian cancer. A loss of BRCA function leads to genomic instability and an elevated risk of tumourigenesis. In the presence of homologous recombination repair deficiency, PARP inhibition causes unrepaired DNA damage in tumour cells. Several factors control homologous recombination, and several genetic defects can impair its function [6]. Therefore, PARP inhibition can affect other tumour cells in addition to BRCA-mutated tumour cells. In turn, PARP overexpression in tumour cells may be a reflection of the high level of DNA damage as well as their increased capacity to stand the effect of DNA-damaging cytotoxic chemotherapy [7]. In theory, PARP expression may be a marker of decreased sensitivity to DNA-damaging environmental effects, such as platinum-based chemotherapy.

Our study aimed to evaluate PARP expression in EOC tissues independently of the BRCA status and to assess its relationship with platinum sensitivity.

## Patients and Methods

### Patient Population

A single institutional, retrospective cohort study was performed at the Department of Obstetrics and Gynecology, the University of Debrecen, on patients with high-grade EOC between 2011 and 2017. All included patients received complete oncotherapy, which covered primary cytoreductive surgery according to the actual European Society of Gynaecological Oncology (ESGO) guidelines and platinum-based adjuvant chemotherapy. In our practice, patients receive six cycles of paclitaxel and carboplatin combined chemotherapy in 3-week cycles (Q3W). Patients participated in follow-up visits, which included physical and performance status examinations, the evaluation of tumour marker levels every 3 months and periodic imaging tests.

### Methods

We collected archived tissue samples of ovarian carcinoma at the Department of Pathology, the University of Debrecen, in agreement with the original approval of the National Ethical Board (ETT 60355–2/2016/EKU). We performed histology and immunohistochemistry according to the standard operating procedures of our diagnostic laboratory. Tissue samples were fixed with formaldehyde (4% in phosphate buffer) for 24 h. The protocol of dehydration and paraffin embedding followed the standard operating procedure. Tissue blocks containing representative tumour tissue were selected and cut to obtain 4 μm thick sections. All evaluated tumour samples originated from the primary site of the tumour.

Immunostaining to highlight PARP protein expression was performed using a Leica Bond MAX Immunostainer (Leica Microsystems, Wetzlar, Germany). For immunostaining, we used a rabbit polyclonal anti-PARP antibody (ab6079 330, Abcam, Cambridge, UK). Tissue sections were deparaffinized and subjected to heat-induced epitope retrieval for 10 min at pH 9.0. The primary antibody was optimal at 1:500 using the Bond Refine-HRP detection system (DS9800, Leica Microsystems, Wetzlar, Germany). We assessed the intensity and distribution of immunostaining by light microscopy (Leica DM2500 microscope, DFC 420 camera, and Leica Application Suite V3 software; Leica). The intensity of specific immunolabelling was determined using a four-grade (0–3+) system, where 0 was equivalent to the total lack of staining and 3+ represented stable and uniform nuclear positivity in the tumour cells. We gave a 2+ score in cases of evident positivity appearing weaker than the maximal intensity.

In contrast, 1+ staining included weak and sometimes highly variable nuclear staining, which was generally different from score 0. An attempt to define the frequency (%) of positive cell nuclei failed due to the heterogeneous composition of the tumour tissues. While most solid tumours evaluated presented with almost no fluctuation in staining intensity (virtually 100%), a significant portion of samples included large nonneoplastic areas (stromal component, inflammation, severe fibrosis) intermixed with the tumour.

Before final analysis, the study population was dichotomized by “any” or “no” PARP expression. The PARP positive group was created from samples, where at least weak staining (1+) was observed by more than 10% of tumor cells. Our theory was that this PARP positive cell population may appear to be sufficient to serve as a starting point for early relapse, selected by PARP induced platinum resistance.

### Data Collection and Statistical Analyses

Clinicopathological features of cases were analysed. The primary endpoint was the progression-free interval between the time of the last chemotherapy cycle to the time of radiologically confirmed relapse. The secondary endpoint was overall survival (OS) at the final analysis to the population dichotomized by “any” or “no” PARP expression.

We calculated descriptive statistics, including the means, medians, and proportions. We used Student’s t test or the Mann-Whitney test and the chi-square test or Fisher’s exact test for the statistical comparisons of continuous or categorical variables, respectively. We generated survival curves using the Kaplan--Meier method and performed Cox proportional hazard regression to identify prognostic variables for progression-free survival (PFS) by multivariate analyses. We used SPSS version 21.0 (IBM Corp., Armonk, NY) for statistical calculations, with significance set at 0.05 and power set at 80%.

## Results

In an attempt to clarify the prognostic relevance of PARP expression, we analysed clinical data of high-grade EOC cases in which PARP immunohistochemistry results were available. Eighty-six patients met the inclusion criteria, and all cases were chemotherapy-naive at the time of PARP immunohistochemistry. The mean age of patients was 57.23 ± 11.13 years. Approximately 81.39% of cases were in an advanced stage (FIGO IIIB-IV). During the histological examination, most of the cases (93.02%) had serous histology, and all cases were high-grade tumours (2-tier grading system). Primary debulking surgery resulted in no residual disease in 43 cases (50%). The median follow-up time was 32.63 months, the median PFS was 13.9 months, and the median OS was 54.5 months (Table 1).

**Table 1:** Characteristics of the Patients

Characteristics	Overall (%)
Number of patients	86 (100%)
Mean age (years)	57,23 (+ − 11,13)
Histological type	
Serous	80 (93,02%)
other	6 (6,98%)
Grade (2-tier)	
High	86 (100%)
Low	0 (0%)
Stage	
Early (FIGO IIIA»)	16 (18,61%)
Advanced (FIGO IIIB«)	70 (81,39%)
Bulky lymph node metastasis	
Yes	24 (27,91%)
No	62 (72,09%)
Primer debulking surgery	
with no residual disease (R0)	43 (50,00%)
with residual disease (R1)	43 (50,00%)
Median follow-up time	32,63 months
No of relapse	63 (73,25%)
Median PFS	13,9 months
No of death	42 (48,83%)
Median OS	54,5 months

**Fig. 1:**
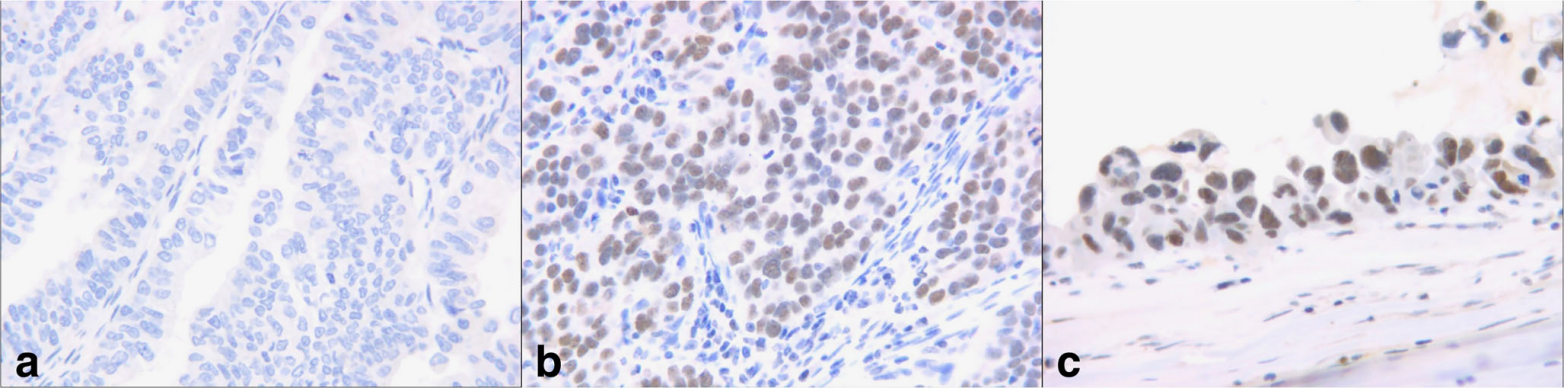
The intensity of specific immunolabelling was determined using a four-grade (0–3+) system, where 0 was equivalent to the total lack of staining (image “**a**”) and 3+ represented stable and uniform nuclear positivity in the tumour cells (image “**c**”). We gave a 2+ score in cases of evident positivity appearing weaker than the maximal intensity (image “**b**”)

We dichotomized the patient population according to PARP expression. A total of 47.68% of cases (*n* = 41) showed no PARP expression, while 52.32% (*n* = 45) showed intermediate or high PARP expression (Fig. 1). PARP-negative and -positive patients showed no significant difference in pretreatment disease characteristics. Among the factors that most influenced survival, the optimal resection rate was 51.22% in the PARP-negative group and 48.89% in the PARP-positive group. The difference was not significant (*p* = 0.49). We did not find any difference in the distribution of early (17.07% vs. 20%) or advanced (82.93% vs. 80%) stage. The rate of relapsed disease was the same among the PARP-positive and -negative subgroups (71.11% and 75.61%, respectively), but the median PFS significantly differed (12.2 vs. 16 months; *p* = 0.01). The number of deaths was 44.44% in the positive group and 46.34%; (*p* = 0.86) in the negative group, but the difference in OS was significant (52 vs. 65 months; *p* = 0.028) (Table 2).

**Table 2:** Characteristics of the Patients after dichomatization by PARP epression

Characteristics			
Variables	PARP positive	PARP negative	*p* value
Number of patients (*n* = 86; 100%)	45 (52,32%)	41 (47,68%)	–
Mean age (years)	57,40 + −10,74 years	57,05 + −11,69 years	0.88
Histological type			
Serous	42/45 (93,33%)	38/41 (92,68%)	0.906
other	3/45 (6,67%)	3/41 (7,32%)	
Grade (2-tier)			
High	45/45 (100%)	41/41 (100%)	1
Stage			
Early (FIGO IIIA»)	9/45 (20,00%)	7/41 (17,07%)	0.72
Advanced (FIGO IIIB«)	36/45 (80,00%)	34/41 82,93%)	
Bulky lymph node metastasis			
Yes	14/45 (31,11%)	10/41 (24,31%)	0.49
No	31/45 (68,89%)	31/41 (75,61%)	
Primer debulking surgery			
with no residual disease (R0)	22/45 (48,89%)	21/41 (51,22%)	0.83
with residual disease (R1)	23/45 (51,11%)	20/41 (48,78%)	
No of relapse	32/45 (71,11%)	31/41 (75,61%)	0.64
Median PFS	12,2 months	16 months	0.01
No of death	20/45 (44,44%)	19/41 (46,34%)	0.86
Median OS	52 months	65 months	0.028

The odds ratio (OR) and 95% confidence interval were calculated to predict the effect of PARP positivity on relapse, PFS less than 12 months, risk of death and survival longer than 32 months. This statistical analysis was performed in stratified form according to the primary tumour resection result (R0 or R1). The results were as follows: PARP positivity did not significant (*p* = 0.638) lower the risk of relapse (OR 0.79; 95% CI 0.30–2.07), but the risk (OR 3.73; 95% CI 1.50–9.26) of short PFS (>12 months) and platinum insensitivity was significantly higher (*p* = 0.004). The risk of death (OR 0.92; 95% CI 0.39–2.16) was slightly lower in the PARP-positive group than the PARP-negative group, but the chance of experiencing improved OS (longer than 32 months) (OR 0.318; 95% CI 0.13–0.76) was significantly lower in this population (*p* = 0.011). To clarify the effect of debulking surgery on this result, we repeated the comparison in the R0 (no macroscopic residual disease) and the R1 (macroscopic residual disease) subgroups of patients. PARP positivity caused a significantly higher risk of short PFS (less than 12 months) (OR 7.916; 95% CI 1.47–42.53; *p* = 0.016) and short OS (less than 32 months) (OR 0.23; 95% CI 0.06–0.83; *p* = 0.025). The results were not significant in other aspects (Table 3).

**Table 3:**
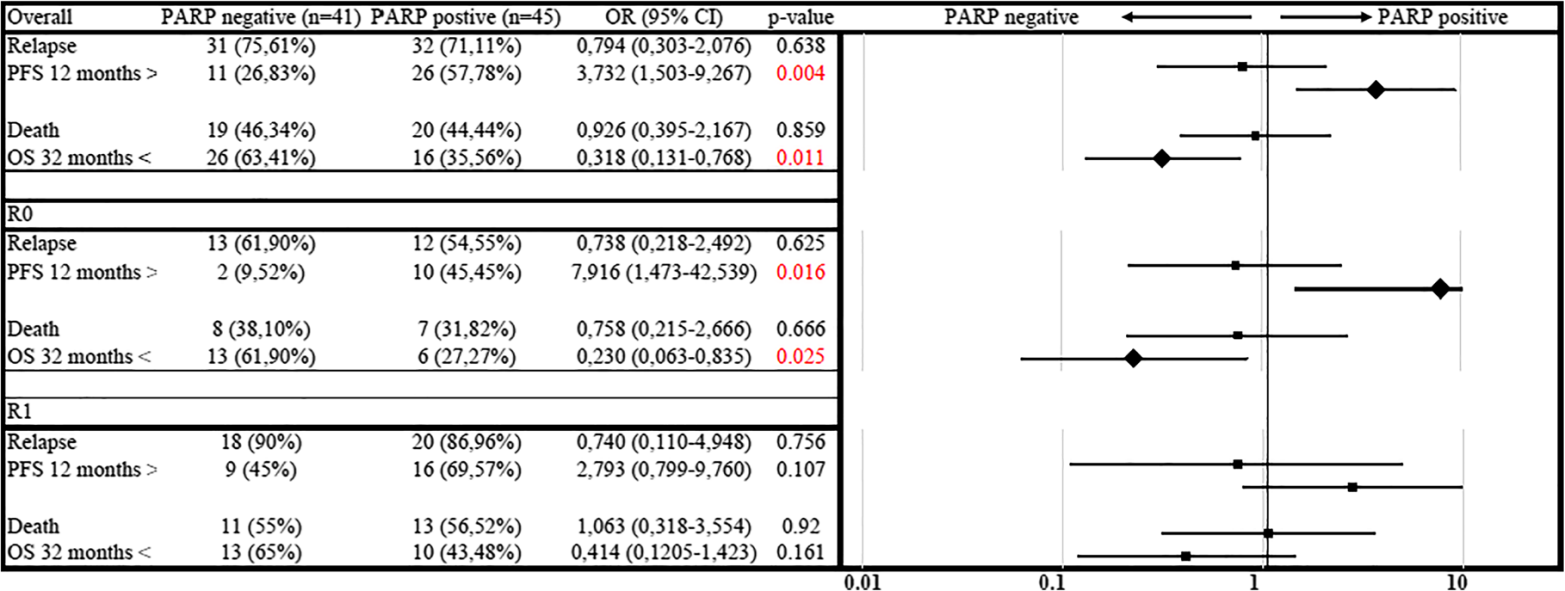
Subgroup analysis of odds ratios for relaps and death

**Fig. 2:**
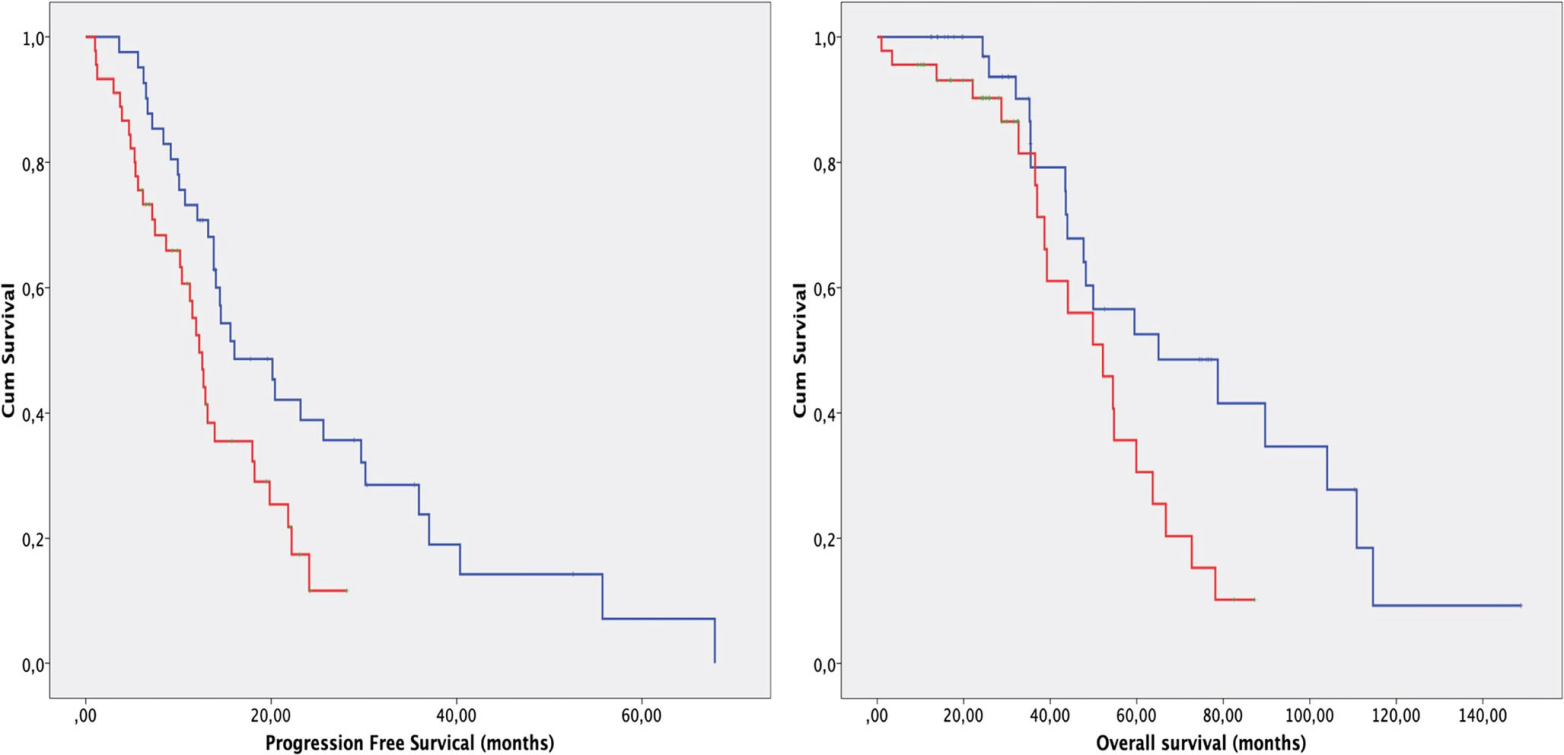
The median PFS among patients in the PARP-negative group was 16 months (interquartile range; IQR 10.7–35.9 months), and the median PFS of patients in the PARP-positive group was 12 months (IQR 6.1–21.8 months). The difference was significant according to the log-rank test (*p* = 0.01). The median OS of patients in the PARP-negative group was 65 months (IQR 43.6–110.8 months), and the median OS of patients in the PARP-positive group was 52 months (IQR 36.9–66.7 months). The difference was significant according to the log-rank test (*p* = 0.028)

In the overall comparison, the analysis of survival curves (Kaplan-Meier curves, log-rank test) showed a substantial difference in PFS and OS between the examined groups. The median PFS among patients in the PARP-negative group was 16 months (interquartile range; IQR 10.7–35.9 months), and the median PFS of patients in the PARP-positive group was 12 months (IQR 6.1–21.8 months). The difference was significant according to the log-rank test (*p* = 0.01). The median OS among patients in the PARP-negative group was 65 months (IQR 43.6–110.8 months), and the median OS of patients in the PARP-positive group was 52 months (IQR 36.9–66.7 months). The difference was significant according to the log-rank test (*p* = 0.28) (Fig. 2).

All evaluated cases were chemotherapy-naive and received the gold standard platinum-based chemotherapy after cytoreductive surgery. Primary PARP immunohistochemistry seems to differentiate between good and poor responders to Taxol-carboplatin chemotherapy. Restricting the analysis to the population dichotomized by “any” or “no” PARP expression resulted in a significant difference in PFS achieved by first-line Taxol-carboplatin chemotherapy.

In conclusion, PARP expression immediately before first-line chemotherapy predicts short PFS, and the lack of PARP expression indicates platinum sensitivity.

## Discussion

The overexpression of PARP may, in theory, lead to reduced sensitivity to platinum-based chemotherapy regardless of the BRCA status. Few studies in the literature have studied the clinical relevance of PARP expression in tumour tissues.

Zhang et al. uncovered a molecular mechanism by which BRCA2 participates in DNA damage repair. They found that the oligonucleotide/oligosaccharide-binding folds (OB-folds) of BRCA2 recognize PAR and mediate the active recruitment of BRCA2 to DNA lesions, which is suppressed by PARP inhibitor treatment. Their findings suggest that PARP plays a role in SSB as well as double-strand break (DSB) repair [8]. In a study published by Bi et al., DNA extracts of BRCA-mutated carrier ovarian cancer tissues and surrounding normal ovarian tissues were compared. The cancer tissues showed decreased DNA methylation in the promoter region of PARP1 compared to that in healthy tissues. Methylation intensity showed an inverse correlation with PARP1 expression. More importantly, the E26 transformation-specific (ETS)-defined CpG sites were significantly less methylated in ovarian cancer samples than in healthy tissues. These findings indicate that hypomethylation around the ETS motifs of the promoter region might contribute to the upregulation of PARP1 expression and the progression of ovarian cancer [9].

Marques et al. screened three cohorts of patients with ovarian cancer, totalling 313 samples. They observed that up to 60% of tumours showed weak PARP1 protein expression. Within a set of 69 cases of high-grade serous ovarian tumours, immunohistochemistry (IHC) showed weak PARP1 expression in 71% of cases. By comparing PARP1 IHC of samples from 31 chemo-naive and 38 chemo-treated cases, they found that the proportion of cases of high PARP1 expression was halved among samples of chemo-treated cases. They also evaluated IHC results in relation to platinum sensitivity, as defined by no disease progression within 6 months of the completion of chemotherapy. Among the 69 cases with evaluable data for comparison, high PARP1 expression was associated with platinum sensitivity (χ^2^ test, *p* < 0.001). Their conclusion is somewhat misleading because the large case numbers were examined only with Western blot protein quantification analyses to compare chemo-naive and chemo-treated tissues, while the prognostic evaluation of high PARP1 expression in terms of PFS <6 months or PFS ≥6 months was assessed in only 69 cases. Among the 69 cases in which IHC results were assessed in relation to platinum sensitivity, there were 56 advanced-stage cases, 35 received neoadjuvant chemotherapy, and 41 cases recurred within a mean follow-up time of 36.6 months.

Our results contradict these findings, since among 41 PARP-negative cases, we found only 5 platinum-resistant cases, while there were 10 platinum-resistant cases among 25 PARP-positive cases. None of our analysed cases had low- or intermediate-grade tumours, and none of them received neoadjuvant chemotherapy. In Marques et al.’s series, 51% of patients received neoadjuvant chemotherapy. Their Western blot analyses confirmed that PARP expression in the tumour tissues is highly influenced by previous exposure to platinum-based chemotherapy; therefore, we believe that our series might be more representative of the native tumour biology and that our results may be more reliable in terms of prognostic evaluation. This conclusion might be further corroborated by the fact that in Marque’s series, only 4 of 69 cases had residual disease upon the completion of surgery. They reported time range upper limits of 84, 72 and 11 months for recurrence, death and follow-up, respectively. Based on these data and those that are typical global in advanced ovarian cancer, the representation of advanced-stage disease in their series might be questionable. We believe that the comparison of continuous survival data of PARP-negative and -positive patients is more accurate than the comparison of categorical survival data, such as case numbers with <6 months and > 6 months [10].

Godoy et al. assessed the expression of PARP in EOC and aimed to correlate its expression with clinicopathologic characteristics. PARP was evaluated using tissue microarray immunohistochemistry in 189 cases of EOC. The authours correlated PARP expression with clinicopathologic variables, including age at diagnosis, stage, grade, histologic type, optimal debulking, PFS, and OS. PARP and p53 expression was observed in 61% and 54% of cases, respectively. PARP-positive tumours are more likely to have a higher grade (*p* = 0.03) and a complete response to initial first-line chemotherapy (*p* = 0.009). The overexpression of PARP in high-grade, advanced-stage tumours indicated that this marker might serve as an indicator of aggressive disease behaviour [11].

Veskimae et al. evaluated PAR concentration in fresh EOC tumour tissue as a surrogate marker for PARP activity. They also evaluated PARP protein expression in archival samples using immunohistochemistry. Their prospective study cohort consisted of 57 fresh tumour samples derived from patients undergoing primary (*n* = 38) or interval debulking (*n* = 19) surgery for EOC and parallel archival paraffin-embedded tumour samples. PARP enzyme activity and PARP staining by IHC showed no correlation (*p* = 0.82). There was an association between high PARP activity and platinum sensitivity in both the entire study population (*p* = 0.022) and in the high-grade subgroup (*p* = 0.017). There was also an association between high PARP activity and improved PFS (32 vs. 14 months, log-rank *p* = 0.009). However, a positive PARP immunostaining pattern was not predictive of improved OS. In conclusion, the authours showed an association between platinum sensitivity and improved PFS with high PARP activity in EOC. They did not confirm an association between PARP IHC and pharmacodynamics assays, and the correlation between PARP IHC results and survival was weak [8].

In contrast, Barnett et al. found that high PARP expression was present in 54% of serous cancers but was not associated with stage or grade. They found that the rate of complete clinical response to primary chemotherapy between cases of low PARP expression (70%) and high PARP expression (71%) did not differ significantly. However, high PARP expression showed a correlation with significantly worse median OS (36 months compared with 43 months, *p* = 0.04, hazard ratio 0.71). They concluded that high PARP expression in serous ovarian cancers is associated with worse OS. Their data are similar to ours and suggest that the evaluation of PARP expression in primary cancers could allow the selective use of PARP inhibitors in patients most likely to respond [12].

In a study by Gan et al., the expression of BRCA1 and PARP1 (in its intact and cleaved (C-PARP1) forms) was immunohistochemically semiquantified in 174 sporadic high-grade serous carcinoma patients. PARP1 expression showed a negative correlation with OS and PFS in patients with a low BRCA1 profile (*p* = 0.04). A multivariate analysis reinforced this finding, revealing PARP1 (*p* = 0.034) as an independent prognostic factor. PARP1 may play an independent role in the response to chemotherapy separate from BRCA gene mutation that is partly due to reduced PARP cleavage [13].

In conclusion, the results of our study suggest that the evaluation of PARP expression can be a useful predictive factor of reduced sensitivity to platinum-based chemotherapy high-grade EOCs. We analysed PARP expression as an all-or-nothing marker. Based on our results, PARP expression immediately before first-line chemotherapy is associated with short PFS, and no PARP expression is significantly associated with platinum sensitivity.
